# Supplementary figures and images for: DNA barcode reference library construction and genetic diversity and structure analysis of Amomum villosum Lour. (Zingiberaceae) populations in Guangdong Province
Source: PeerJ. 2021 Oct 20;9:e12325. doi: 10.7717/peerj.12325 (PMC8541303; doi:10.7717/peerj.12325)

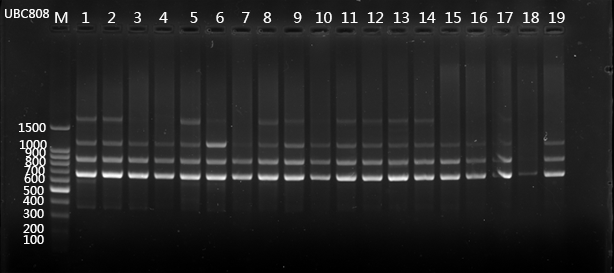


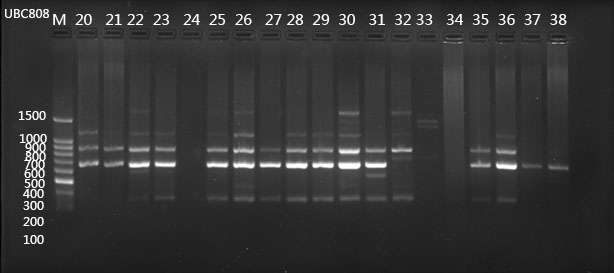


**Fig. S1 ISSR-PCR electrophoretic maps of *A. villosum* populations with primer UBC808**

Supplement: Supplemental Information 1 [file peerj-09-12325-s001.docx]
