# Supplementary material for: DNA barcode reference library construction and genetic diversity and structure analysis of Amomum villosum Lour. (Zingiberaceae) populations in Guangdong Province
Source: PeerJ. 2021 Oct 20;9:e12325. doi: 10.7717/peerj.12325 (PMC8541303; doi:10.7717/peerj.12325)
Supplement: Supplemental Information 2 [file peerj-09-12325-s002.docx]

**Table S1 The primers and PCR conditions of five DNA barcodes.**

| primers | | base（5'-3'） | amplification conditions |
| --- | --- | --- | --- |
| ITS2 | 2F | ATGCGATACTTGGTGTGAAT | 94℃，5min  94℃，30s；56℃，30s；72℃，45s. 35 circles  72℃，10min |
|  | 3R | GACGCTTCTCCAGACTACAAT |  |
| *psbA-trnH* | fwd PA | GTTATGCATGAACGTAATGCTC | 94℃，4min  94℃，30s；55℃，1min；72℃，1min. 35 circles  72℃，10min |
|  | rev TH | CGCGCATGGTGGATTCACAATCC |  |
| *matK* | 3F_KIM | CGTACAGTACTTTTGTGTTTACGAG | 94℃，1min  94℃，30s；52℃，20s；72℃，50s. 35 circles  72℃，5min |
|  | 1R_KIM | ACCCAGTCCATCTGGAAATCTTGGTTC |  |
| *rbcL* | 1F | ATGTCACCACAAACAGAAAC | 95℃，4min  94℃，1min；55℃，1min；72℃，2min. 38 circles  72℃，7min |
|  | 724R | TCGCATGTACCTGCAGTAGC |  |
| ITS | 5F | GGAAGTAAAAGTCGTAACAAGG | 94℃，5min  94℃，1min；50℃，1min；72℃，1min30s. 30 circles  72℃，7min |
|  | 4R | TCCTCCGCTTATTGATATGC |  |
